# Numerical Study of Hydrodynamic Forces for AFM Operations in Liquid

**DOI:** 10.1155/2017/6286595

**Published:** 2017-07-26

**Authors:** Tobias Berthold, Guenther Benstetter, Werner Frammelsberger, Rosana Rodríguez, Montserrat Nafría

**Affiliations:** ^1^Deggendorf Institute of Technology, Dieter-Görlitz-Platz 1, 94469 Deggendorf, Germany; ^2^Universitat Autònoma de Barcelona (UAB), Bellaterra, 08193 Barcelona, Spain

## Abstract

For advanced atomic force microscopy (AFM) investigation of chemical surface modifications or very soft organic sample surfaces, the AFM probe tip needs to be operated in a liquid environment because any attractive or repulsive forces influenced by the measurement environment could obscure molecular forces. Due to fluid properties, the mechanical behavior of the AFM cantilever is influenced by the hydrodynamic drag force due to viscous friction with the liquid. This study provides a numerical model based on computational fluid dynamics (CFD) and investigates the hydrodynamic drag forces for different cantilever geometries and varying fluid conditions for Peakforce Tapping (PFT) in liquids. The developed model was verified by comparing the predicted values with published results of other researchers and the findings confirmed that drag force dependence on tip speed is essentially linear in nature. We observed that triangular cantilever geometry provides significant lower drag forces than rectangular geometry and that short cantilever offers reduced flow resistance. The influence of different liquids such as ultrapure water or an ethanol-water mixture as well as a temperature induced variation of the drag force could be demonstrated. The acting forces are lowest in ultrapure water, whereas with increasing ethanol concentrations the drag forces increase.

## 1. Introduction

**Figure 1:**
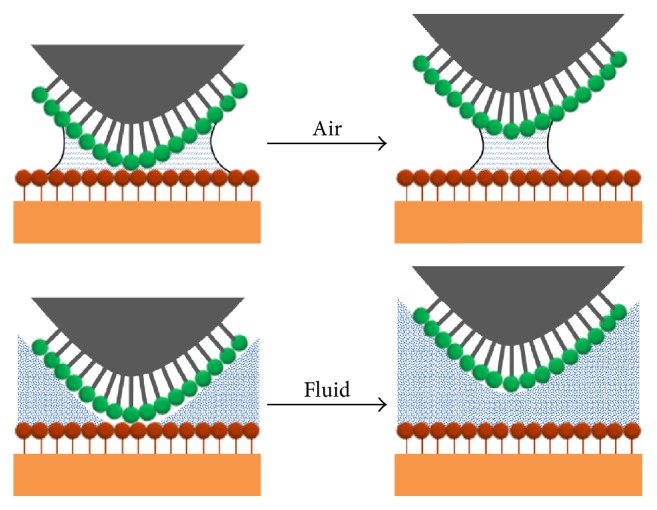
Comparison of the tip-sample interaction during separation in air and in fluid environment.

Since the invention by Binnig et al. in 1986 [1], atomic force microscopy (AFM) is finding increasing use in the characterization of chemical surface modifications [2, 3] to achieve a chemical mapping of the surface in the nanoscale. This advanced method is called Chemical Force Microscopy (CFM) [3, 4] and is based on sensing the interaction forces between a chemical modified tip and the sample surface. The measurement environment plays an important role for the performance of CFM because any attractive or repulsive forces influenced by the measurement environment could obscure the molecular forces.  Figure 1 displays the comparison of the tip-sample interaction influenced by the measurement environment. The water meniscus due to the water layer present in air strongly influences the pull-off force caused by the capillary effect. The pull-off forces obscure the molecular adhesion forces under surveillance between the modified tip and the sample surface as they are two to three magnitudes larger [5, 6]. A liquid environment (Figure 1) can eliminate this capillary effect and thus the detrimental effect of the pull-off forces and hence enables the measurement of molecular adhesion forces one or two orders of magnitude less than is possible in air [5, 7].

In CFM the adhesion forces, representing the molecular interaction forces, strongly depend on the chemical modification of the AFM tip and the sample surface [3]. For a CH_3_ modified tip and a highly hydrophobic sample surface, adhesion forces up to 15 nN can be measured in a water environment [3, 8].

Beside the increased sensitivity for detecting adhesive forces, the liquid environment also improves the investigation of very soft sample surfaces by applying very low forces. Therefore, in recent literature AFM measurements of biological samples were undertaken in liquid environment [9–11].

The mechanical behavior of the moving AFM cantilever depends on density and viscosity of the ambient medium. A liquid, such as ultrapure water, possesses substantially different values of density and viscosity than air. At standard environmental conditions of 293 K and 1013 hPa, the density of air *ρ*_air_ is 1.2044 kg/m^3^ and the dynamic viscosity *μ*_air_ is 1.8140 · 10^−5^ Pa·s. The density *ρ*_water_ of ultrapure water is 999.615 kg/m^3^ and the dynamic viscosity *μ*_water_ is 1.009347 · 10^−3^ Pa·s. The much higher density and viscosity of the liquid affect the force measurements by hydrodynamic drag forces [11–13]. Various researchers stated that the influence of this effect is more significant for cantilever tip velocities above a few *μ*m/s [11–15].

The general influence of a viscous fluid on the cantilever movement was already reported by Sader [16]. He introduced an analytical model to determine the frequency response of rectangular and circular cantilever beams operated in a viscous fluid. However, beside the limitation to specific cantilever geometries, Sader's model does not consider the drag force dependence on the cantilever-sample distance which is very important for force measurements [13].

In their study, Méndez-Méndez et al. [11] introduced a numerical model to predict the hydrodynamic forces acting on a V-shaped tip for velocities up to 105 *μ*m/s and varying cantilever-sample distances. They show that the usage of different fluids significantly influences the drag force acting on the cantilever and confirm that the drag force dependence on tip speed is essentially linear in nature.

In recent literature, for the investigation of very soft samples or especially organic samples, the Peakforce Tapping (PFT) mode [20] was introduced to control vertical forces in the range of some tens of pico-Newtons [10, 21]. Additionally, PFT enables the mapping of nanomechanical properties [20, 22]. This advanced characterization technique also provides advantages for the investigation of chemical modification by CFM because this dynamic operation mode enables a complete chemical mapping of the surface [23, 24] and is not just limited to applying individual force distance curves at distinct measurement points.

In contrast to the hydrodynamic drag forces for tip velocities up to 105 *μ*m/s researched by Méndez-Méndez et al. [11], for PFT performed in an aqueous solution, the cantilever tip is operated in a sinusoidal *z*-movement with an oscillation frequency of usually 1 kHz and oscillation amplitudes ranging from 50 nm to 300 nm. Based on these parameters, the maximum cantilever tip velocity in the fluid can vary from 314 *μ*m/s to even 1885 *μ*m/s. Therefore, other researchers stated that the hydrodynamic forces in a fluidic environment can be as high as 10–20 nN [21].

This magnitude of parasitic hydrodynamic forces may distort the outcomes and could limit the control of the vertical forces significantly. Amo and Garcia [25] indicated that the ringing in the oscillation caused by the hydrodynamic forces introduces an error in the determination of the adhesion force. The accurate extraction of the adhesion force and the detection of the peak force are related to the zero line of the force distance curve during the stationary state, where the tip is not in contact with the sample. An offset of this zero-force line caused by hydrodynamic drag forces may distort the adhesion forces and the desired peak forces.

Consequently, further research is necessary to investigate the hydrodynamic drag forces acting on the cantilever in PFT operation mode in liquid environment. The present work addresses these needs and provides a numerical model to investigate the hydrodynamic drag forces for different cantilever geometries and varying fluid conditions for tip speeds associated with PFT measurements. Therewith, the measurement setup can be optimized by minimizing parasitic drag forces and by reducing experimental uncertainties for a more precise force control regarding studies of soft samples in liquids.

## 2. Model Parameter

**Figure 2:**
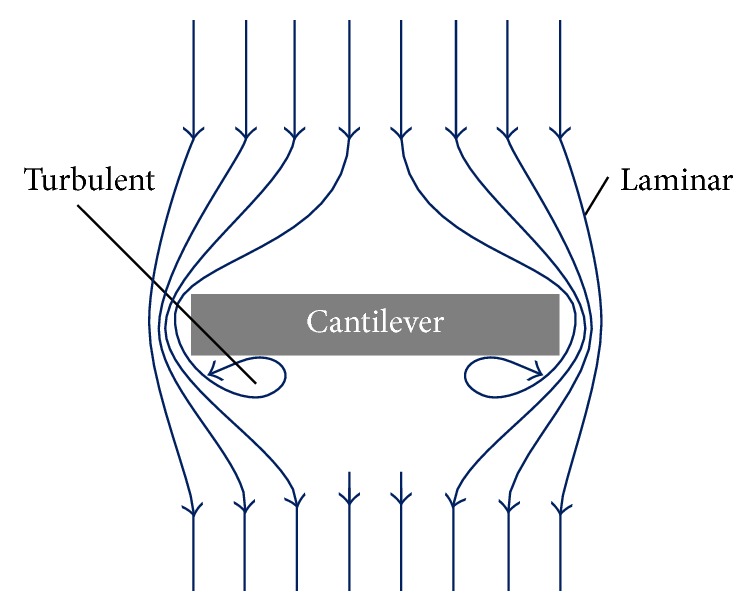
Principle of the fixed cantilever flowed by a fluid. Laminar and turbulent fluid flow effects are marked.

For the simulation of the hydrodynamic effect, the cantilever movement in the fluidic environment is interpreted as a fixed body flowed by a fluid. This principle, schematized in Figure 2, assumes that the cantilever movement in the fluidic environment is identical to a fluid circulating around a fixed cantilever. Since the relative velocity between cantilever and liquid remains the same, both approaches are valid for the simulation of the hydrodynamic force. Figure 2 shows two different flow effects. Laminar flow occurs if the fluid passes the cantilever without turbulences and accordingly turbulent flow is described as a stream subject to disturbances.

The laminar as well as the turbulent flow could affect the drag force acting on the AFM cantilever during its movement and therefore both must be considered in the numerical model. In this study the simulation was performed by using the Comsol CFD (computational fluid dynamics) model which is based on the Reynolds-Averaged Navier Stokes (RANS) formulation for incompressible fluids. The RANS formulation represents the conservation of momentum and can be written as follows:

(1) ρ∂u→∂t+u→·∇u→=−∇p+∇μ∇u→+∇u→T+∇μt∇u→+∇u→T−23ρKt,

where $\vec{u}$ represents the fluid velocity in each spatial direction, *p* is the fluid pressure, *ρ* and *μ* are the fluid density and the fluid dynamic viscosity, respectively, *μ*_*t*_ represents the turbulent viscosity, and *K*_*t*_ is the turbulent kinetic energy. *T* is defined as the notation for a transposition. Since the fluids used in this work are known to be incompressible Newtonian fluids, the fluid pressure *p* is defined to be constant over the whole volume.

Additionally, the continuity equation representing the conservation of mass is solved and described as

(2) $$\begin{array}{c} \rho \nabla \vec{u}=0. \end{array}$$

For the evaluation of the turbulent viscosity *μ*_*t*_ an additional transport model must be introduced. A very versatile model is the *K*-epsilon (*K*-*ε*) turbulence model [26, 27]. Combined with the RANS equations, this model can be used to simulate both laminar and turbulent flow effects.

**Figure 3:**
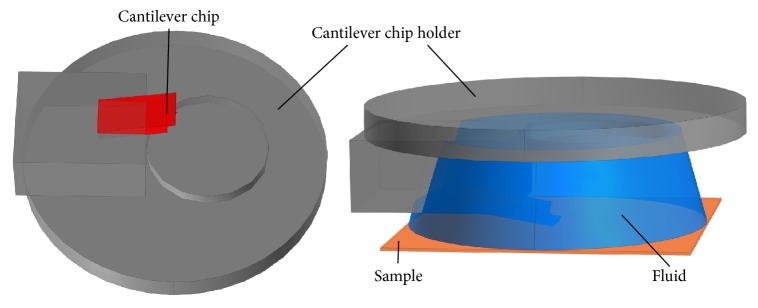
Geometrical model of the cantilever holder (DECAFMCH from Bruker AXS) with a mounted AFM probe (Figure 4) (left) and probe holder with mounted probe immersed in a fluid on top of a sample surface (right).

For the AFM measurements, a special cantilever holder for fluid operations is used.  Figure 3 presents the geometrical model developed for the simulation of the fluid dynamics according to the cantilever holder DECAFMCH from Bruker AXS. The geometry in Figure 3 is modeled for realistic conditions during the AFM scan in fluid. The probe tip is completely immersed in the liquid, which completely covers the sample surface at the area of interest.

Since the distance between the AFM cantilever and the sample surface influences the drag forces [11, 12], the hydrodynamic contributions due to the presence of the sample surface were considered in the simulation model. The cantilever tip end was positioned 500 nm above the sample surface. This distance was chosen because, in preliminary studies, it could be observed that a further decrease of this distance down to 25 nm resulted in an increase of the drag forces by only 1%.

### 2.1. Cantilever Model

**Figure 4:**
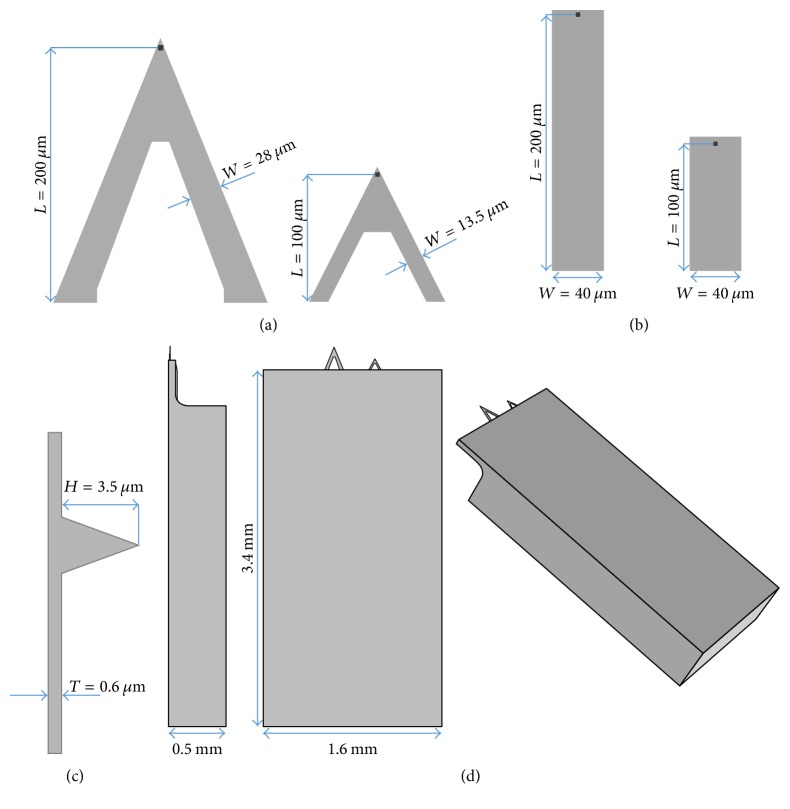
(a) Triangularly shaped cantilever with a length *L* of 200 *μ*m and 100 *μ*m; (b) 200 *μ*m and 100 *μ*m long rectangularly shaped cantilever; (c) cantilever thickness and AFM tip geometry identical for all geometries; (d) main components considered for the cantilever model consisting of the support chip which carries the Si_3_N_4_ cantilever (here triangular).

In this study, commercially available cantilever types with chemically modified tips (ST-PNP from Nanoandmore GmbH) are considered. All cantilevers are made of silicon nitride (Si_3_N_4_). The shape is either triangular or rectangular.  Figure 4 shows the geometrical dimensions of the different cantilever including the supporting chip.

Beside the geometry of the cantilever material, parameters such as Young's modulus *E* are very important because they strongly influence the stiffness *k* and the deflection of the cantilever. In case of Young's modulus *E* an exact value for the cantilever material is not specified by the manufacturer. In contrast, the average stiffness or spring constant *k* of the cantilevers is provided by the manufacturer. Accordingly, the triangular cantilevers possess spring constants of 0.08 N/m and 0.32 N/m and the rectangular cantilevers of 0.06 N/m and 0.48 N/m for 200 *μ*m and 100 *μ*m cantilever length, respectively. An overview of all cantilever dimensions and their averaged spring constants provided by the manufacturers is summarized in Table 1.

However, these values may vary due to some manufacturing tolerances. For the fluid dynamics the effective values for *k* as well as for Young's modulus *E* are very important because, based on Hooke's law,

(3) $$\begin{array}{c} F=k\cdot \Delta z, \end{array}$$

and the force *F* acting on the cantilever is directly dependent on the effective spring constant *k* and the corresponding cantilever deflection Δ*z*. Analytical models for calculating the spring constant introduced by Sader et al. [28, 29] are mostly limited to standard cantilever geometries such as rectangular or V-shaped ones and are not suitable for the triangular cantilevers examined in this work. However, since Young's modulus *E* is an important material parameter of the cantilevers (Figure 4), the relation of Young's modulus *E* and the respective spring constant was evaluated by applying a force in the range of 1 nN to the tip. Young's modulus *E* of the cantilever material was varied in the range of 200–300 GPa.

**Figure 5:**
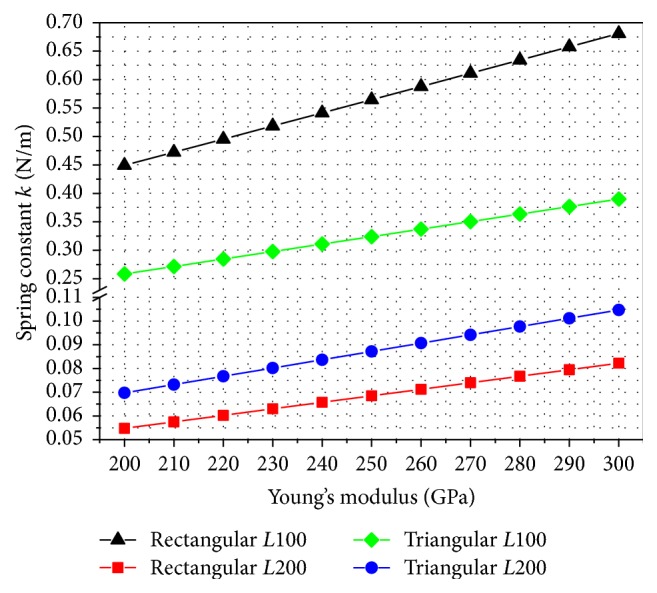
Relationship between spring constant *k* and Young's modulus *E* for the rectangular and triangular cantilever with a length *L* of 200 *μ*m and 100 *μ*m, respectively.

Due to the achieved deflection of the cantilever the corresponding spring constant *k* was calculated using (3).  Figure 5 illustrates the computed values in a diagram with *k* as a function of *E*. Since the material for all cantilevers is the same, the material properties specified in the simulation model were kept constant. Therefore, Young's modulus for Si_3_N_4_ used as cantilever material was defined to be 250 GPa and the corresponding spring constant introduced in Table 2 was evaluated based on the results outlined in Figure 5. The resulting spring constants for the rectangular cantilevers are in close agreement with the values of *k* = 0.0675 and *k* = 0.54 for the 200 *μ*m long and the 100 *μ*m rectangular cantilever obtained using Sader's analytical model [29]. However, the relation of spring constant and Young's modulus *E* achieved by the finite element approach was used in our simulation model to calculate the hydrodynamic drag forces because it is assumed that this approach considers the different cantilever geometries more accurately.

### 2.2. Fluid Model

**Figure 6:**
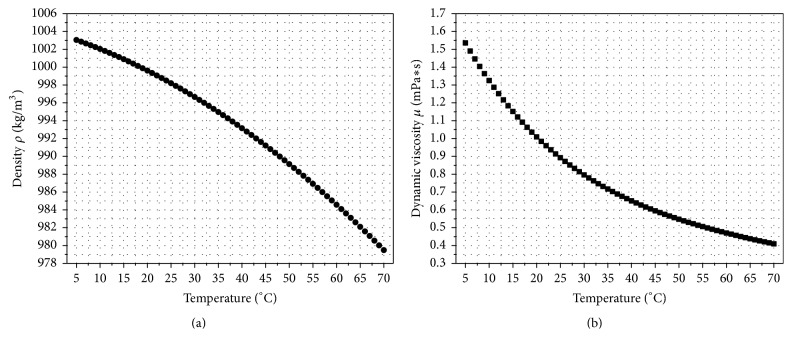
Temperature versus density (a) and dynamic viscosity (b) of water derived from the Comsol built-in material library and [17].

Since the hydrodynamic drag forces are strongly affected by the fluid parameters, influencing factors on these parameters must be considered in the simulation model. The main parameters of the fluid influencing the hydrodynamic forces are the density *ρ* and the dynamic viscosity *μ*. Since the fluid temperature can be influenced by the ambient environment or the energy dissipation of the AFM equipment, the temperature dependence of the density *ρ* and the dynamic viscosity *μ* was considered. The relationship between temperature and density as well as temperature and dynamic viscosity for ultrapure water is indicated in Figure 6. Both curves exhibit the significant temperature influence on the fluid conditions and were taken into account for the fluid model.

**Figure 7:**
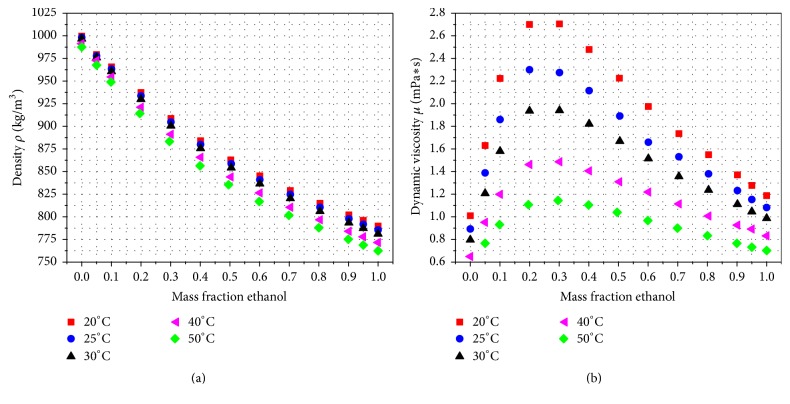
Change of the density *ρ* (a) and the dynamic viscosity *μ* (b) in relation to the mass fraction of ethanol in an ethanol-water mixture at different temperatures based on [18, 19].

Beside the usage of ultrapure water, Kokkoli and Zukoski [30] as well as Yaacobi and Ben-Naim [31] have shown in their studies that mole fractions of ethanol (EtOH) up to 0.2 can increase the strength of hydrophobic interaction forces between probe tip and sample. Additionally, they stated that a higher ethanol mole fraction in the ethanol-water mixture results in a significant lowering of the interaction forces, though by mixing ethanol with water liquid properties such as the density *ρ* and the dynamic viscosity *μ* may be changed significantly. The diagrams in Figure 7 relate the mass fraction of ethanol in an ethanol-water mixture to the density *ρ* and the dynamic viscosity *μ* of the resulting solvent at different temperatures.

Obviously, adding ethanol to an ethanol-water mixture causes a very opposed trend for density and viscosity of the mixture. Particularly, for the interesting range below a mass fraction of 0.2, Figure 7 shows that while density is reduced slightly, dynamic viscosity is increased dramatically with rising EtOH content. Additionally, Figure 7 displays the temperature dependence. While the temperature dependence of the density *ρ* is comparatively low, the dynamic viscosity *μ* changes significantly with temperature.

By using the fluid parameters presented in Figures 6 and 7, a very comprehensive simulation model covering the important fluid conditions for AFM measurements can be implemented. The highest drag forces may be expected for the maximum relative velocity of cantilever and fluid. This is independent of whether the model of Figure 2, resting cantilever and moving fluid, with which the force calculations are performed, or real measurement conditions, resting liquid and moving cantilever, is considered. The maximal cantilever velocity *v*_max_ for the model can be deduced from real measurement conditions by

(4) $$\begin{array}{c} v_{max}=z_{max}\cdot 2\pi f, \end{array}$$

where *z*_max_ represents the maximal amplitude of the sinusoidal cantilever movement and *f* is the oscillation frequency. Thereby, the force deflecting the cantilever induced by the movement in the liquid medium can be calculated.

## 3. Results and Discussion

**Figure 8:**
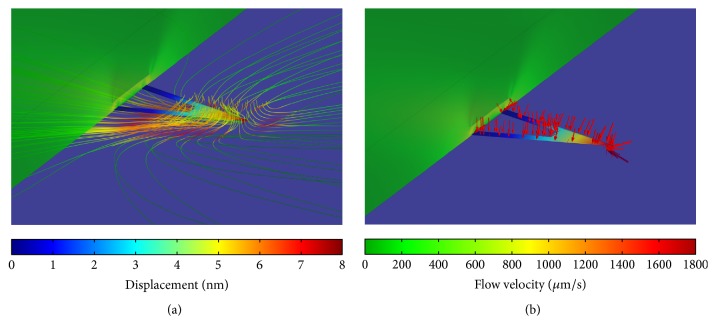
Simulation result (*z*_max_ = 100 nm, *f* = 1 kHz) around the cantilever area showing in (a) the flow velocity around the cantilever by streamlines as well as the deflection of the cantilever and in (b) also the deflection of the cantilever as well as the pressure acting on the cantilever because of the fluid flow highlighted by red arrows.

The introduced simulation model can evaluate the deflection of the cantilever caused by the liquid environment.  Figure 8 presents the simulated flow velocity (a) around the cantilever and the pressure (b) acting on the cantilever, both combined with the resulting cantilever deflection. The colors of the cantilever shape illustrate the respective deflection of the cantilever according to the displacement color scale. It is obvious that the tip end of the cantilever experiences the highest bending. The streamlines depicted in Figure 8(a) represent the fluid flow around the cantilever. The associated flow velocity of the streamlines is indicated by the respective color scale. It can be concluded that the resulting velocity variation is induced by the resistance to flow of the cantilever. Due to this resistance, the liquid flow results in pressure acting on the cantilever which is highlighted by the red arrows in the Figure 8(b) and this pressure causes the deflection of the cantilever. Upon these results, the drag force can be calculated by using the spring constant which was evaluated in the previous section.

### 3.1. Evaluation of the Sensor Dynamics Simulation Model

For an evaluation of the present model appropriate for PFT operations presented in this work, our results were confirmed by comparing with published data of other researchers. Janovjak et al. [12] quantified hydrodynamic drag forces as a function of pulling speed using the scaled spherical model of Alcaraz et al. [13].

**Figure 9:**
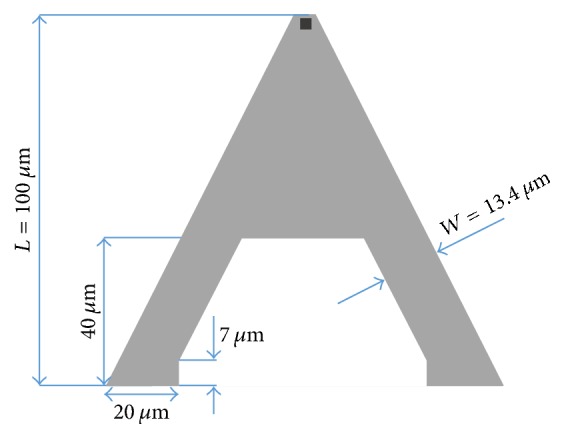
Cantilever model OTR4 Olympus with a thickness of 0.4 *μ*m according to the data in [11, 12].

Méndez-Méndez et al. [11] introduced a numerical 3D model to predict the hydrodynamic drag force in measurements undertaken in fluids. In both works, the hydrodynamic drag force obtained for a small OTR4 Olympus V-shaped cantilever in water was presented.  Figure 9 shows the respective cantilever model with exact geometric dimensions.

**Figure 10:**
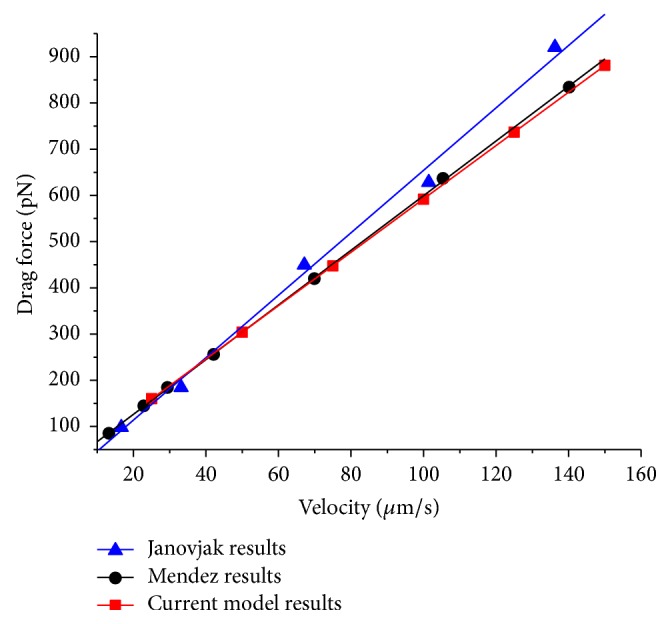
Comparison of the FEM simulation results with the numerical predictions published by Méndez-Méndez et al. [11] and Janovjak et al. [12].

Figure 10 compares the results of the present model with the results of the other studies. The linear dependence of drag force and tip velocity is clearly visible. The predictions of the present model accord very well with the results of Méndez-Méndez et al. [11]. The maximum deviations of both models are about 2%. In contrast, Figure 10 shows that results of Janovjak et al. [12] differ significantly. The deviations to the present model rise over 10%. This difference can be explained by the cantilever shape which is not considered in the scaled spherical model introduced by Alcaraz et al. [13] and applied by Janovjak et al. [12] for quantifying the hydrodynamic drag forces. This model is based on a drag force model of a sphere very close to a plane wall and for the more complex cantilever geometry, two empirical coefficients representing the effective size of the cantilever and the effective cantilever tip height are introduced.

The agreement of our model with the model introduced by Méndez-Méndez et al. [11] confirms the suitability of the presented model to quantify the hydrodynamic drag forces for different cantilever geometries and varying fluid conditions for tip speeds associated with PFT. Due to the linear relationship of tip speed and drag force, the presented model can be validated by comparing with results associated with much lower tip speeds and extrapolated to the PFT tip speeds. The difference of these models to the scaled spherical model of Alcaraz et al. [13] shows the influence of the cantilever shape on the drag force.

For an additional evaluation of the present simulation model by experimental data, individual approach curves with lower tip speeds can be suggested to extract the drag forces at a tip-sample distance of 500 nm. For experimental data with PFT tip speeds, the measurement equipment would have to be updated to bypass the background subtraction algorithm [21] and measurement data of the *z*-sensor must be extracted.

### 3.2. Sensor Dynamics in Ultrapure Water

For AFM fluid imaging applications, the frequency for the probe tip movement was fixed to 1 kHz. The amplitude of the cantilever oscillation can be changed in the range of 50 nm up to 300 nm. Therefore, the flow velocity in the simulation model was varied within this amplitude range with respect to (4).

**Figure 11:**
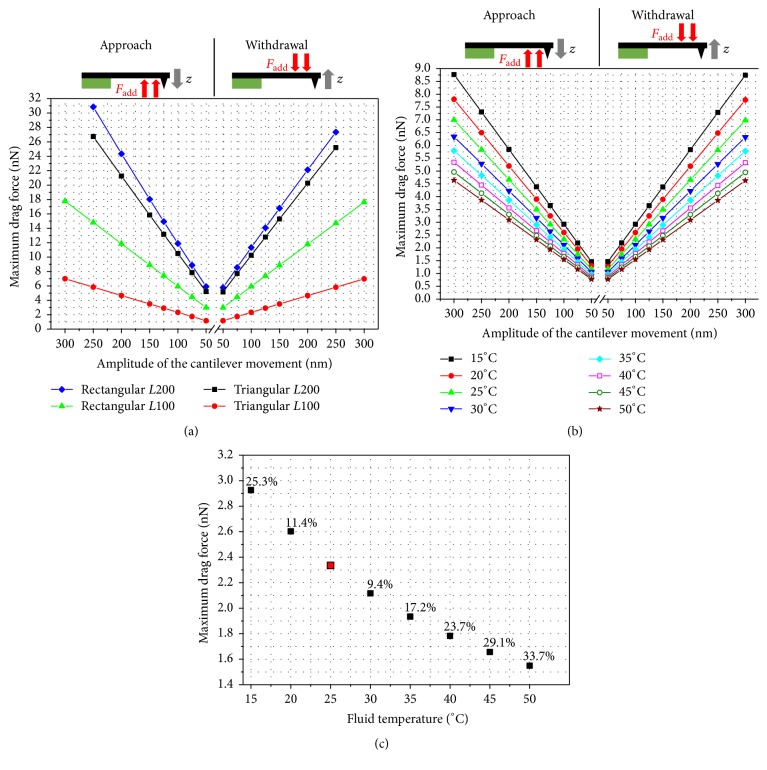
Maximum hydrodynamic drag force during operation in ultrapure water for a tip-sample distance of 500 nm versus oscillation amplitude during the approach and withdrawal movement of (a) different AFM cantilevers at a temperature of 25°C; (b) the 100 *μ*m long triangular cantilever at various fluid temperatures. (c) Force variation induced by the fluid temperature for the 100 *μ*m long triangular cantilever at an amplitude of 100 nm indicating the percentage change based on 25°C.

Figure 11(a) presents the hydrodynamic drag force in relation to the oscillation amplitude for the different cantilevers under investigation. The drag force acting on the cantilevers changes significantly with the amplitude and displays a linear behavior.

The maximum drag force for the triangular cantilever is much smaller than for the rectangular cantilever geometry. It may be interpreted that triangular cantilevers provide an improved flow behavior. Shorter cantilevers cause smaller drag forces than longer cantilevers. This can be explained by the fact that with cantilever length the effective area presented to the liquid is increased which causes increased drag forces and vice versa. In detail, the respective top-side areas are 2747 *μ*m^2^ and 9723 *μ*m^2^ for the 100 *μ*m and 200 *μ*m long triangular cantilevers and 4200 *μ*m^2^ and 8200 *μ*m^2^ for the 100 *μ*m and 200 *μ*m long rectangular cantilevers, respectively. Notwithstanding that the cantilever areas of the shorter cantilevers correlate with the smaller drag forces presented in Figure 11(a), it can be stated that the drag force differences of the 200 *μ*m long cantilevers do not follow the trend of their areas. Despite the larger area of the 200 *μ*m long triangular cantilever, the shape provides better flow characteristics. These results show that both the cantilever area and the cantilever shape are important parameters influencing the fluid dynamics during the motion.

As can be seen in Figure 11(a), the cantilevers are also subject to different drag forces for approach and withdrawal movement. These differences vary from 0.28% and 0.79% for the 100 *μ*m triangular cantilever and the 100 *μ*m rectangular cantilever to 6.09% and even 12.80% for the 200 *μ*m triangular cantilever and the 200 *μ*m rectangular cantilever, respectively. The general difference can be explained by the tip geometry increasing the drag force during the approach movement. Since the tip geometry is the same for all cantilevers, the significant difference can be explained by the different spring constants of the cantilevers and the cantilever shapes influencing the flow characteristics of the liquid.

As an important outcome, it is worth noting that the 200 *μ*m long cantilevers provide a much lower relative difference in drag force for triangular versus rectangular geometries compared to 100 *μ*m long cantilevers. This correlates with the difference of the respective spring constants illustrated in Table 2. Overall it is obvious that the triangular cantilever with a length of 100 *μ*m features the lowest drag force during the approach movement as well as during the withdrawal.

The computed drag forces are much higher than the values calculated for the evaluation of the simulation model in Figure 10. The tip speed during the PFT operation ranges from 1885 *μ*m/s for an amplitude of 300 nm to 314 *μ*m/s for an amplitude of 50 nm and these values are much higher than the velocities considered in Figure 10. For this reason, the drag forces achieved for the PFT conditions are much higher. Comparted to these results, Schillers et al. [21] also mentioned that the hydrodynamic forces can be as high as 10–20 nN. As can be seen in Figure 11(a), it can be confirmed that such force values are in the scope of this work depending on cantilever type and length as well as amplitude of cantilever movement. As an important conclusion, for PFT operations in liquid environment triangular cantilever geometry and short cantilever are recommended. Consequently, for the further examinations presented in this study the triangular cantilevers with a length of 100 *μ*m were considered.

The outcomes in Figure 11(a) were achieved for operation in ultrapure water at 25°C. These conditions are close to normal ambient air. However, during operation the AFM equipment and thus the liquid environment become warmer due to energy dissipation of the equipment. In Figure 11(b), the influence of the fluid temperature on the drag force is plotted for a 100 *μ*m long triangular cantilever. As expected from Figure 6, increasing fluid temperature lowers the drag force significantly. By examining the results in more detail, we could figure out that the relative variation induced by temperature change is identical for each amplitude value.  Figure 11(c) presents the drag force over the fluid temperature for ultrapure water and a 100 *μ*m long triangular cantilever operated at an oscillation amplitude of 100 nm. The labels of the data points represent the percentage change with respect to the force at a temperature of 25°C. An increase from 25°C to 30°C reduces the drag force by 9.4% and a further increase to 35°C decreases the drag force by 17.2%. It can be shown that the relation between drag force and fluid temperature is closely linked to the change of dynamic viscosity (Figure 6), because the percentage change of dynamic viscosity and drag force are very similar for the temperature range shown in Figure 11(c) and both curves can be approximated by quadratic fit lines. Thus, it is evident that the main driving force for the relation of drag force and temperature in ultrapure water is the dynamic viscosity of the medium.

Due to Figures 11(b) and 11(c), it can be concluded that a slight temperature increase reduces the drag force significantly and may thus influence AFM measurement results. Therefore, to start AFM fluid imaging procedures after equipment and cantilever holder including the respective liquid have reached thermal equilibrium is recommended, which is for normal AFM measurements in fluids in the range of 25°C to 35°C. The corresponding forces were calculated up to a temperature of 50°C (Figure 11(b)). Such temperature ranges could be reached by a purposely applied additional sample heating. In this case, it must be considered that the evaporation rate of the liquid is increased and the liquid volume must be controlled frequently to ensure stable measurement conditions.

### 3.3. Sensor Dynamics in an Ethanol-Water Mixture

**Figure 12:**
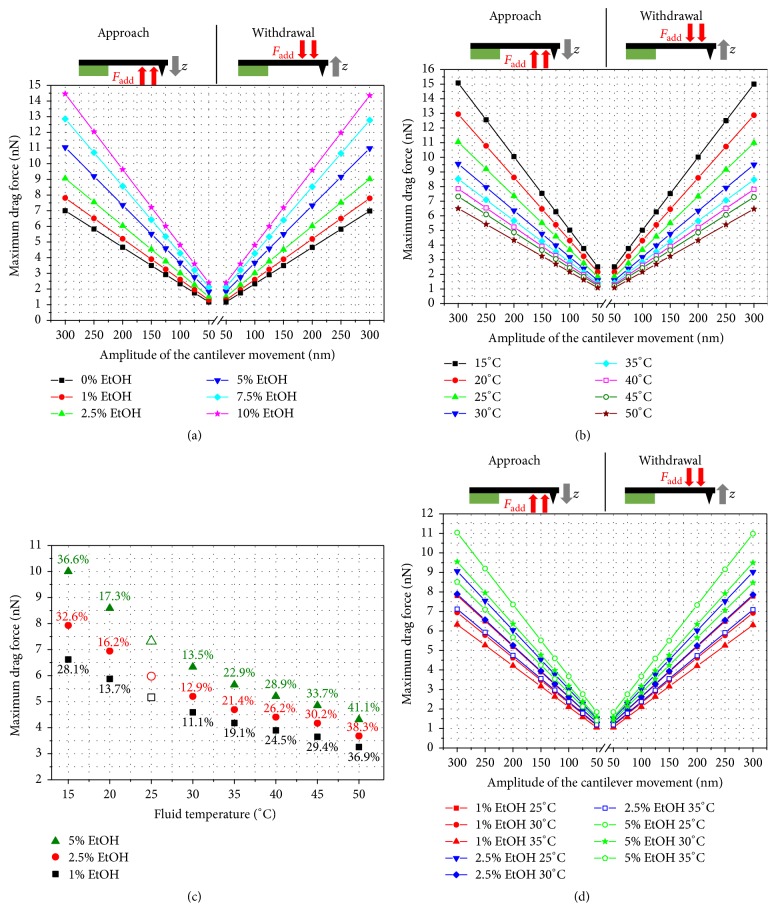
Maximum hydrodynamic drag force during operation of the 100 *μ*m long triangular cantilever in an ethanol-water mixture for a tip-sample distance of 500 nm versus oscillation amplitude during the approach and withdrawal movement for (a) varying EtOH concentrations up to 10% at a temperature of 25°C, (b) 5% EtOH at fluid temperatures from 15°C up to 50°C based on Figure 7, and (d) 1%, 2.5%, and 5% EtOH concentrations with fluid temperatures ranging from 25°C to 35°C (100 *μ*m long triangular cantilever). (c) Force variation induced by the fluid temperature for different EtOH concentrations (100 nm triangular cantilever, 200 nm oscillation amplitude). The data point labels indicate the relative change for the respective concentration to a fluid temperature of 25°C.

The results presented so far were achieved for ultrapure water as fluid medium. Other researchers [30, 31] have shown that the use of an ethanol-water mixture may be advantageous, for example, by strengthening the interaction forces between tip and sample. The drag forces for the 100 *μ*m long triangular cantilever and varying ethanol content over the amplitude of cantilever movement are represented in Figure 12(a). It is obvious that an increased ethanol concentration increases the resulting drag forces significantly. Even a comparatively low ethanol concentration of 10% doubles the hydrodynamic forces.

In their experimental investigation, Yaacobi and Ben-Naim [31] mentioned that the molecular interaction forces are amplified for an ethanol concentration in the range from 3% to 20%. In addition, the experimental data of Kokkoli and Zukoski [30] show strengthening of the attraction and adhesion of two hydrophobic surfaces for an ethanol concentration *x*_EtOH_ ≤ 0.05. Since both research articles [30, 31] provide recommended EtOH concentrations in the range of 5% or even lower to amplify interaction forces and since low EtOH fractions, and thus reduced hydrodynamic forces, are preferable, this range is considered for further investigations. The detailed examination of this EtOH range in Figure 12(a) shows that 1% EtOH increases the drag forces by approximately 11.6%, 2.5% EtOH raises the drag forces to 29.3%, and 5% EtOH even provides 57.5% higher forces. Compared to the density and dynamic viscosity of these EtOH ranges (Figure 7), it can be determined that due to the significant variation the dynamic viscosity is the main parameter influencing the drag force.

Since the values in Figure 12(a) are related to environment conditions of 25°C, analogous to the application of pure water, the temperature impact was analyzed for the ethanol-water mixture used as sensor fluid.  Figure 12(b) shows the influence of the temperature on the drag force from 15°C up to 50°C for a 5% ethanol-water mixture. The significant decrease of the drag force caused by elevated temperatures is evident and was already indicated by the significant influence of the temperature on the dynamic viscosity *μ* (Figure 7). A temperature rise to 35°C enables the decrease of the drag forces by 22.9% and an increase to 50°C lowers the value even by 41.1%. Since 5% and lower EtOH concentrations are recommended [30], the temperature impact by using EtOH fractions below 5% was analyzed. Like the previous investigations for the operation in ultrapure water, we observed that the relative drag force variation induced by temperature change is identical for each amplitude value.  Figure 12(c) presents the drag force over the temperature of the ethanol-water mixture for 1%, 2.5%, and 5% EtOH. The labels of the data points represent the percentage change with respect to the drag force at a temperature of 25°C.  Figure 12(c) clearly illustrates that the temperature induced deviation of the drag force varies for the different EtOH concentrations. Additionally, the main driving force for the temperature influence can be identified to be the significant decrease of the dynamic viscosity shown in Figure 7.

It was already mentioned that a temperature of 50°C could only be reached by purposely using an additional sample heating and that the most important temperature range due to the self-heating of the equipment goes from 25°C to 35°C. For this reason, Figure 12(d) presents the temperature influence on the drag force in the self-heating temperature range for the preferable EtOH concentrations of 5% and below.

Different colors allocate the corresponding ethanol content. The highest drag forces correspond to 5% EtOH at 25°C and the lowest forces correspond to 1% EtOH at 35°C. Between these extreme values, Figure 12(d) shows that the drag force range for each ethanol concentration may overlap depending on the temperature.

AFM imaging in liquids is frequently used to characterize molecular interaction forces or to investigate soft samples in biological applications. The forces under surveillance are comparatively small compared to drag forces acting on the cantilever imposed by the liquid measurement environment and the tip movement. Thus these drag forces disturb the measurements. The presented model and the achieved results reveal that the hydrodynamic drag forces can be determined exactly for each individual measurement setup and for various fluid properties used for AFM fluid imaging. These results can be used to optimize the measurement setup and thus improve the experimental significance and validity.

## 4. Conclusion

In this work a numerical integrated model was presented that is able to provide accurate predictions of the hydrodynamic drag forces present in AFM fluid imaging applications in general and in chemical force measurements in particular. The presented results included a wide range of cantilever types, cantilever oscillation amplitudes, fluid types, and fluid temperatures. The dynamic viscosity could be shown to be the most important fluid parameter influencing cantilever movement in ultrapure water or in an ethanol-water mixture. The numerical 3D model employed was verified by comparing the predicted drag forces with published results of other researchers. The findings of this section confirmed that drag force dependence on tip speed is essentially linear in nature.

The numerical results could show that the triangular cantilever geometry is preferable for AFM measurements in fluid because it provides significant lower drag forces than the rectangular cantilever geometry. Beside the examination of different cantilevers and their oscillation amplitudes, the influence of the used fluid medium such as ultrapure water or an ethanol-water mixture could be demonstrated. The results showed that ultrapure water provided the lowest drag forces, whereas with increasing ethanol concentration the drag forces increase. In addition, the presented fluid temperature dependence on the drag force clarified that besides the self-heating of the equipment an additional heat source could be used for a further improvement of the parasitic hydrodynamic forces. By operating the 100 *μ*m long triangular cantilever with standard parameters (frequency = 1 kHz and oscillation amplitude = 100 nm) in ultrapure water at a temperature of 35°C, the hydrodynamic drag force can be stated to be 1.93 nN.

## Figures and Tables

**Table 1:** Summary of the geometrical dimensions of the cantilevers introduced in Figure 4 and their spring constant provided by the manufacturers.

Cantilever shape	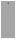	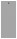	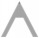	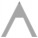
Length *L* [*µ*m]	100	200	100	200
Width *W* [*µ*m]	40	40	13.5	28
Thickness *T* [*µ*m]	0.6	0.6	0.6	0.6
Tip height *H* [*µ*m]	3.5	3.5	3.5	3.5
Spring constant *k* [N/m]	0.48	0.06	0.32	0.08

**Table 2:** Evaluated spring constant *k* of the cantilevers for Young's modulus *E* of 250 GPa.

Cantilever shape	Length *L* in *µ*m	Relation *k* = *f*(*E*)	Spring constant *k* in N/m
Rectangular	200	*k* = *E* · (2.746 · 10^−4^ m)	0.0687
Triangular	200	*k* = *E* · (3.494 · 10^−4^ m)	0.0874
Rectangular	100	*k* = *E* · (2.305 · 10^−3^ m)	0.5763
Triangular	100	*k* = *E* · (1.311 · 10^−3^ m)	0.3278
